# Germanium as a scalable sacrificial layer for nanoscale protein patterning

**DOI:** 10.1371/journal.pone.0195062

**Published:** 2018-04-06

**Authors:** Bochao Lu, Michel M. Maharbiz

**Affiliations:** 1 UC Berkeley-UCSF Graduate Program in Bioengineering, University of California, Berkeley, CA, United States of America; 2 Electrical Engineering and Computer Science Department, University of California, Berkeley, CA, United States of America; 3 Chan Zuckerberg Biohub, San Francisco, CA, United States of America; Brandeis University, UNITED STATES

## Abstract

We demonstrate the use of germanium (Ge) films as water-soluble features that allow the patterning of proteins onto surfaces with commonly used organic solvents. This technique is scalable for manufacturing and is compatible with nano- and microfabrication processes, including standard lithography. We use Ge as a sacrificial layer to mask and protect areas of the substrate during surface functionalization. Since Ge dissolves in 0.35% hydrogen peroxide (H_2_O_2_) in water but not in organic solvents, Ge can be removed after patterning without significantly affecting protein activities. In this paper, we present examples of protein patterning with two different techniques. We show that 50 nm thick Ge layers can be completely removed in 10 min without residues and, importantly, nanoscale resolution and misalignment can be achieved with conventional photolithography equipment. Both biotin and streptavidin maintain ~80% and >50% activity after 10 min and 360 min incubation in 0.35% H_2_O_2_, respectively. Lastly, the process can be used to functionalize sidewalls with proteins, a capability of recent interest for cell-cell adhesion studies.

## Introduction

As researchers miniaturize biosensors and microfluidic devices down to submicron scales[1–3], high-resolution biomolecule conjugation compatible with these processes has become desirable. Several protein patterning techniques have been demonstrated, including gasket-based patterning[4,5], microcontact printing (μCP)[5–9], and dip pen lithography (DPL)[10–12]. Gasket-based patterning is the easiest and arguably most popular protein patterning approach in academic work. In this approach, removable polymer gaskets, usually made of polydimethylsiloxane (PDMS), are used as a mask on the conjugation surface to localize surface functionalization reagents. Since alignment is usually performed manually, it is difficult to precisely align the polymer gaskets with existing surface structures. This method is suitable for low resolution and low throughput research projects, but is difficult to scale up. DPL and μCP are both based on the contact between small probe tips with surface chemistry reagents and a substrate surface. μCP uses PDMS stamps with micron[6,9] or, more recently, submicron features[8] to pattern functionalization reagents onto a substrate. This method exhibits higher resolution and throughput than that of gasket-based methods, but the alignment between desired protein patterns and existing features is limited by the alignment tool. DPL involves using an atomic force microscopy (AFM) tip to trace out the functionalization patterns and can achieve nanoscale resolution with submicron alignment, but usually requires custom AFM systems and is thus difficult to scale up for bulk manufacturing.

To achieve high resolution, precise alignment and bulk manufacturability, engineers have long turned to photolithography techniques to pattern surface moieties. For example, lithographically patterned gold films can be functionalized with thiol groups to pattern proteins onto gold surfaces selectively[13–15]. However, gold thin films may have strong interference with fluorescent signals, so this process may not be suitable for some cases requiring a fluorescent readout. Another common technique involves depositing a sacrificial layer, such as common photoresists, to occlude a region of the surface where conjugation is unwanted, and subsequently dissolve the sacrificial layer with a biocompatible solvent[16,17,3]. However, photoresists can be problematic for surface chemistries that require organic solvents, since photoresists dissolve quickly in most of the commonly used organic solvents (e.g. ethanol, acetone, isopropyl alcohol (IPA) and dimethyl sulfoxide (DMSO)). For example, silane is commonly used for silica surface functionalization, but due to the hydrolysis of silane groups in aqueous solution, an organic solvent like ethanol or DMSO is preferred for silane dissolution in order to maintain the activity of silane groups. A sacrificial layer which will dissolve in a biocompatible solution but not in commonly used organic solvents would enable precise surface conjugation across a wider range of surface chemistries. While there are alternatives to photoresist such as using a dissolvable metal as a sacrificial layer in protein lift off[18], the dissolution rates of these metals are low at neutral pH, they are difficult to deposit, and some are flammable.

Here, we demonstrate a simple, inexpensive and manufacturable protein patterning technique with submicron resolution and only nanoscale misalignment using germanium (Ge), a semiconductor material, as a sacrificial layer for a lift-off process. This method is suitable for creating patterns of a target material on a surface using a sacrificial material. Ge has been studied for decades in the field of microelectromechanical systems (MEMS) and has been used as a sacrificial layer due to its solubility in aqueous solutions[19,20]. Ge dissolves slowly in aqueous solution, but is not affected by most organic solutions[21]. Germanium is first oxidized into germanium dioxide by oxidizing agents (e.g. dissolved oxygen molecules or aqueous hydrogen peroxide (H_2_O_2_)). H_2_O_2_ accelerates the oxidation of Ge since it is a much stronger oxidizing agent than oxygen molecules. The spontaneous dissolution of germanium dioxide in water (containing some oxidizer) exposes more Ge for further oxidation until complete dissolution of the Ge sacrificial layer occurs[19,20].

In brief, our process is as follows: A thin layer of germanium is first patterned. The entire surface (containing germanium and non-germanium coated surfaces) is then functionalized and the germanium is then dissolved away, leaving biomolecules only on the surfaces that originally were uncoated by germanium. The whole process is scalable and easy to apply in any microfabrication lab, as it relies on standard thin film techniques. In addition, germanium and its dissolution products are biocompatible, which enables the use of this technique for both *in vitro* and *in vivo* devices[22].

In this paper, we report protein patterning results from two different Ge deposition processes, E-beam evaporation and low temperature chemical vapor deposition (LPCVD). Since E-beam evaporation is more directional (more material deposited on horizontal surface than on vertical sidewalls) and LPCVD is more conformal (same film thickness on both vertical and horizontal surfaces), LPCVD protects vertical walls from biomolecule attachment while E-beam evaporation enables sidewall functionalization, which is a topic of recent interest[23]. Both biotin and streptavidin surface activity were characterized with various incubation periods in 0.35% H_2_O_2_. To our knowledge, this study is the first demonstration of protein nanoarray fabrication with Ge as a sacrificial layer.

## Experimental methods

Substrate features were first patterned onto p-type silicon wafers by conventional photolithography techniques. Deep ultraviolet (DUV) photoresist (Dow UV210-0.6) was spun at 7000 rpm for 420 nm final thickness, exposed in ASML 5500/300, developed in MF26A for 1 min and hard baked for 2 h at 120°C (all DUV photoresists were patterned with these conditions unless otherwise indicated). Wafers were then etched in a Lam TCP 9400SE Etcher to provide 120 nm-deep alignment markers, which enabled alignment of protein patterns with substrate features in subsequent germanium patterning. In this paper, we etched 500 nm deep square wells into the substrate in a Lam Etcher system to demonstrate alignment accuracy and sidewall functionalization. After well etch, we deposited a layer of high-temperature oxide (HTO) in a TYTAN Diffusion Furnace System at 900°C. HTO was not necessary for our protein patterning technique with a Ge mask, but provided a blank surface with binding sites for silane chemistry. If other surface chemistries are used, the HTO step can be skipped and the Ge process applied after substrate fabrication.

**Fig 1:**
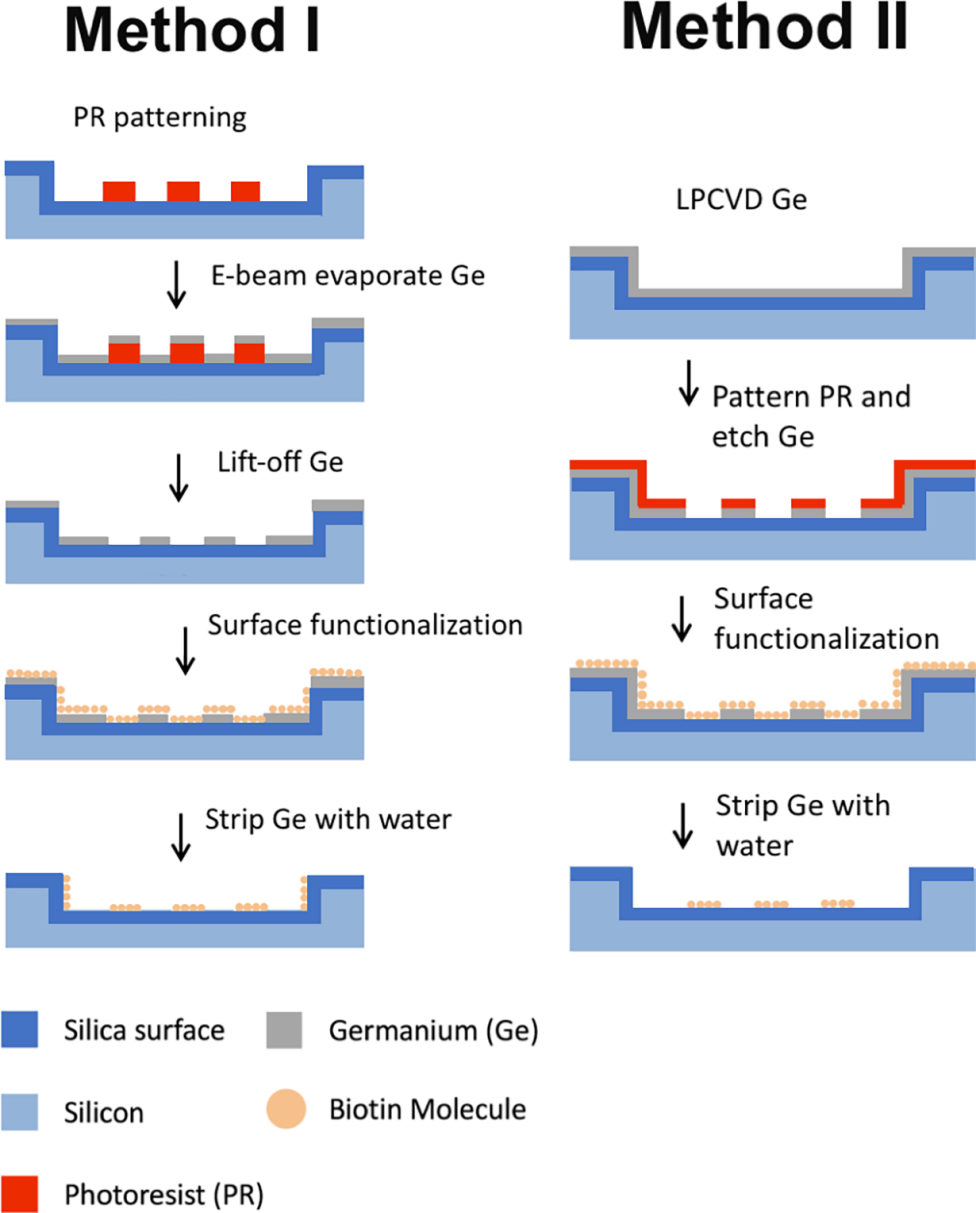
Fabrication process overview. Left) 50 nm Ge layer is patterned with E-beam evaporation and a lift-off process. Vertical sidewalls are functionalized along with horizontal openings. Right) 50 nm Ge layer is patterned with LPCVD and etching. Vertical sidewalls are completely covered and not exposed to functionalization solution. After Ge patterning and surface chemistry, Ge layer is dissolved in 0.35% H_2_O_2_ and the chip is ready to use.

There are two Ge fabrication methods presented in this paper (Fig 1). In method 1, we used an E-beam evaporation and lift-off process to pattern Ge. DUV photoresist (Dow UV210-0.6) was spun at 1480rpm to form a 900nm sacrificial layer and patterned to cover the areas to be functionalized. In this work, we patterned micron scale squares inside the previously etched square wells as a demonstration. 50 nm thick germanium was evaporated on top of the patterned photoresist in a CHA E-beam Evaporator at 10^−6^ Torr base pressure. The surface conjugation areas were then exposed by stripping photoresist in 1-methyl-2-pyrrolidone (NMP). In method 2, we deposited Ge first with LPCVD and then etched away Ge in surface functionalization areas. 50 nm thick germanium was deposited in a TYTAN Diffusion Furnace System at 340°C after depositing a layer of 2 nm amorphous silicon as an adhesive layer between HTO and Ge. Then, 420 nm DUV photoresist was patterned over the germanium. Germanium exposed through the photoresist was completely etched away in a Lam TCP 9400SE Etcher to open windows for protein patterning in the next step. The plasma etching of germanium had high selectivity against HTO so that HTO also worked as a stop layer during germanium etching. After Ge patterning with either method, we brought the substrates in contact with 25 mg/ml silane-PEG-biotin (Nanocs, New York, NY) in ethanol or dimethyl sulfoxide (DMSO) and incubated for 30 min at room temperature. After washing away excess silane solution, wafer substrates were immersed in deionized (DI) water with 0.35% hydrogen peroxide to strip Ge. After the dissolution of germanium, wafers with biotin patterns were washed in DI water and blown dry with nitrogen, followed by storage in a 4°C fridge for future use.

Germanium dissolution rate as a function of H_2_O_2_ concentration, temperature and pH had been well characterized in literature[24]. Our previous work also demonstrated that nanometer-thick Ge dissolved in low concentration H_2_O_2_ in minutes while no significant dissolution discovered in all tested organic solvent[21]. Before surface conjugation with silane chemistry, dissolution of Ge was tested in 0.35% H_2_O_2_ and surfaces were imaged with a Gemini Scanning Electron Microscope (SEM) before and after dissolution. Substrates were first patterned with 25 μm by 25 μm square wells that were etched 500 nm deep, followed by 100 nm HTO deposition. A layer of 50nm Ge was deposited in an LPCVD furnace and 3 μm by 3 μm squares were etched inside the square wells. The etching recipe had a 20 s overetch after the 50nm Ge etch to ensure all Ge and amorphous Si on top of the HTO was etched away. Substrates were then imaged in SEM to identify the Ge surface versus oxide surface. After immersion in 0.35% H_2_O_2_ for either 10 min or overnight at room temperature, substrate surfaces were imaged by SEM again to characterize the surface after Ge dissolution. It was very easy to distinguish oxide from Ge in SEM, since the oxide surface was smoother and brighter than the Ge surface which had micron-size grains.

Prior to functionalization, 25 μm by 25 μm and 42 μm by 42 μm square wells were etched 500 nm deep into the silicon wafer, followed by 100 nm HTO deposition. All samples were separated into two groups. Group 1 and 2 were fabricated and functionalized using Method 1 and 2, respectively (Fig 1). 3 μm x 3 μm and 5 μm x 5 μm squares patterned inside 25 μm x 25 μm and 42 μm x 42 μm square wells (16 squares per well) were biotinylated using silane-PEG cross linker (see *Fabrication*). Wafers were then incubated with 1% bovine serum albumin (BSA) blocking solution for 1 hour at room temperature to prevent nonspecific binding. Streptavidin conjugated with Alexa-Fluor 647 (Strep-647) was used to fluorescently label the biotinylated surface by incubating 10 μg/ml Strep-647 in 1X PBS with substrate surfaces for 30min at room temperature, after which Ge was removed by 10 min-immersion in 0.35% H_2_O_2_ with mild stirring. Since Ge is dissolved after Strep-647 labelling, it was not strictly necessary to incubate in blocking solution; the step was carried out to be certain that Strep-647 bound to biotin specifically (as streptavidin is known to bind nonspecifically to surfaces). We did not find significant signal decrease due to oxidation in H_2_O_2_, so all Strep-647 labeling was done before Ge dissolution (with the exception of the experiment to test biotin activity in H_2_O_2_). Fluorescent images were taken using a Nikon Eclipse Ti fluorescent microscope after washing and blowing dry.

The resolution of proteins patterned with Ge hard masks was limited by the photolithography process and exposure tools. In order to show that the limit was attributable to lithography limits, we pushed the resolution of our protein patterning down to 250 nm, the limit of our ASML model. For this experiment, 500 nm deep square wells were not etched before HTO deposition, since a 500nm height difference disrupted the uniformity (even distribution of photoresists) of DUV photoresist coating (420 nm thick) and affected submicron lithography. Thus, protein nanoarrays were fabricated on flat silicon wafers with 100 nm HTO and Method 2 was used to pattern 50 nm Ge film with 1 μm x 1 μm, 500nm x 500nm, 300nm x 300nm and 250nm x 250nm openings for conjugation. Biotinylation of nanoarrays was performed as per *Fabrication.* Strep-647 was then used as described above to label biotin molecules on the surface. Fluorescent images were taken with a Carl Zeiss Elyra SR.1 Super Resolution Microscope in order to resolve the 250 nm squares.

As the concentration of H_2_O_2_ used for Ge dissolution was very low, both biotin and streptavidin surface exhibited sufficient activity for the following experiments. Biotin-conjugated chips were fabricated as above. Biotin activity was demonstrated by incubating biotin conjugated chips in 0.35% H_2_O_2_ for various periods from 10 to 360 min at room temperature. For streptavidin-activity tests, biotin conjugated chips were firstly incubated with 100ug/ml non-fluorescent streptavidin solution for overnight to get streptavidin surfaces. Streptavidin conjugated chips were immersed in 0.35% H_2_O_2_ for different incubation periods as per biotin groups. (Note, sufficient H_2_O_2_ solution and a sealed the container should be used for long incubations to prevent drying. If the H_2_O_2_ solution dries or too much water evaporates during incubation, neither biotin nor streptavidin survive). After H_2_O_2_ incubation, biotin and streptavidin chips were blocked in 1% BSA for 1h at room temperature before brought in contact with Strep-647 and Atto-488 Biotin (Biotin-488), respectively. Fluorescent images were then taken with a Nikon Eclipse Ti fluorescent microscope after washing and blowing dry.

## Results & discussion

**Fig 2:**
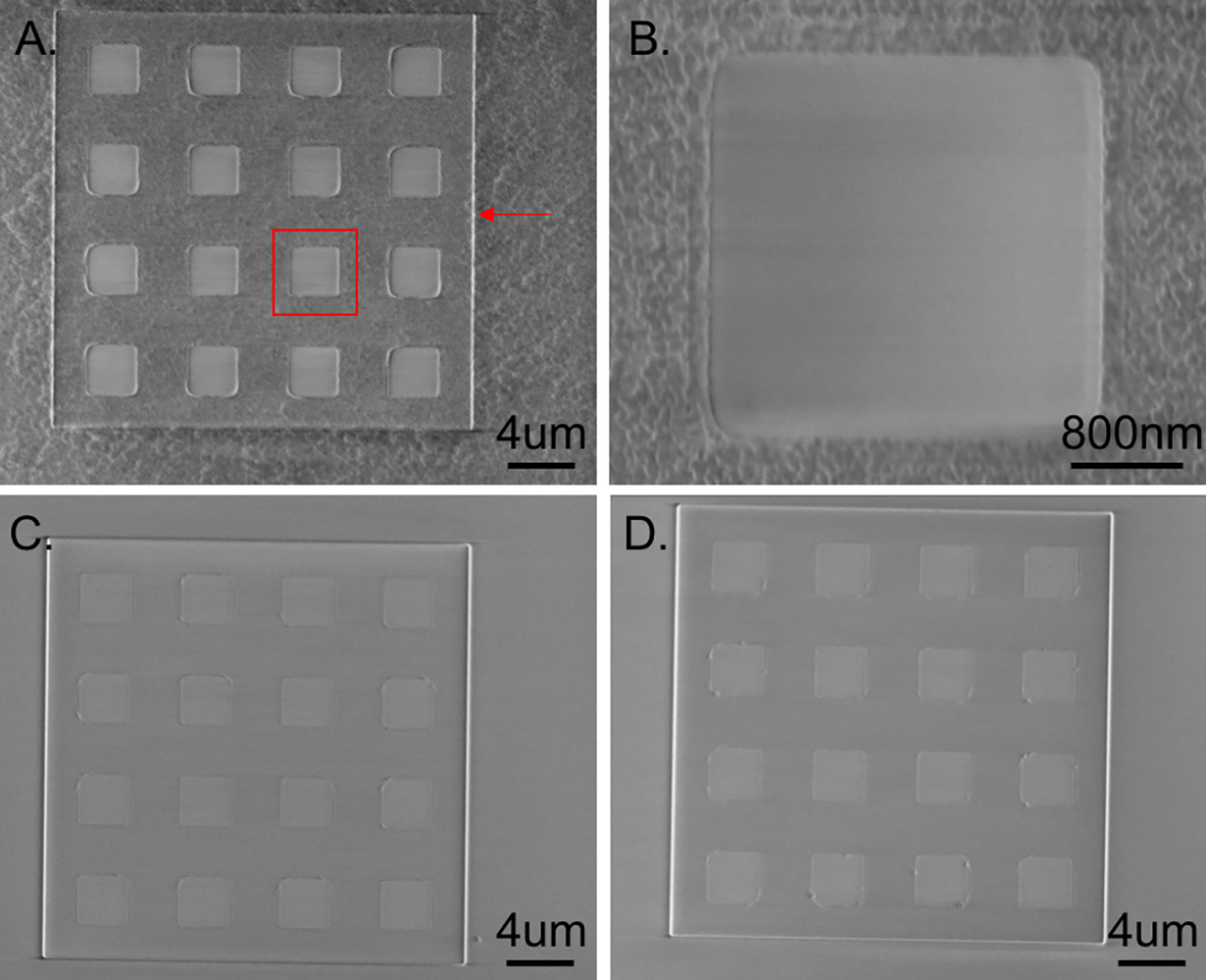
Germanium dissolution. A) SEM image of 50 nm Ge films deposited by LPCVD before dissolution. Red arrow indicates the edge of a 25 μm by 25 μm square well, etched 500nm deep. B) Higher magnification of red box in A). C&D) SEM images of Ge layers after 10min C) and overnight D) incubation in 0.35% H_2_O_2_ at room temperature. Ge was completely dissolved in 10min with no residues left. Faint square outlines are due to the over-etch process in the Ge etching recipe and thus the adhesive amorphous Si layer between Ge and HTO is etched away.

Germanium dissolved rapidly in 0.35% H_2_O_2_ at room temperature. As shown in Fig 2A & 2B, each 25 μm x 25 μm well had sixteen 3 μm by 3 μm squares for functionalization. The exposed oxide area was smoother than the Ge layer, for which micro-scale grains were evident. After 10 min incubation in 0.35% H_2_O_2_, it was very clear that all Ge grains were completely removed and overnight incubation didn’t remove more material (Fig 2C & 2D). Any residual Ge on the oxide surface would be very obvious in SEM. Thus, a 10min incubation in 0.35% H_2_O_2_ was sufficient to remove a 50nm Ge sacrificial layer. After Ge dissolution, 3 μm by 3 μm squares were still visible under SEM (Fig 2C & 2D). This arose because the additional over-etch step in the Ge etching process etched away the 2 nm Si layer in those 3 μm by 3 μm conjugation regions, while the 2 nm Si layer elsewhere did not dissolve in H_2_O_2_ during Ge dissolution.

**Fig 3:**
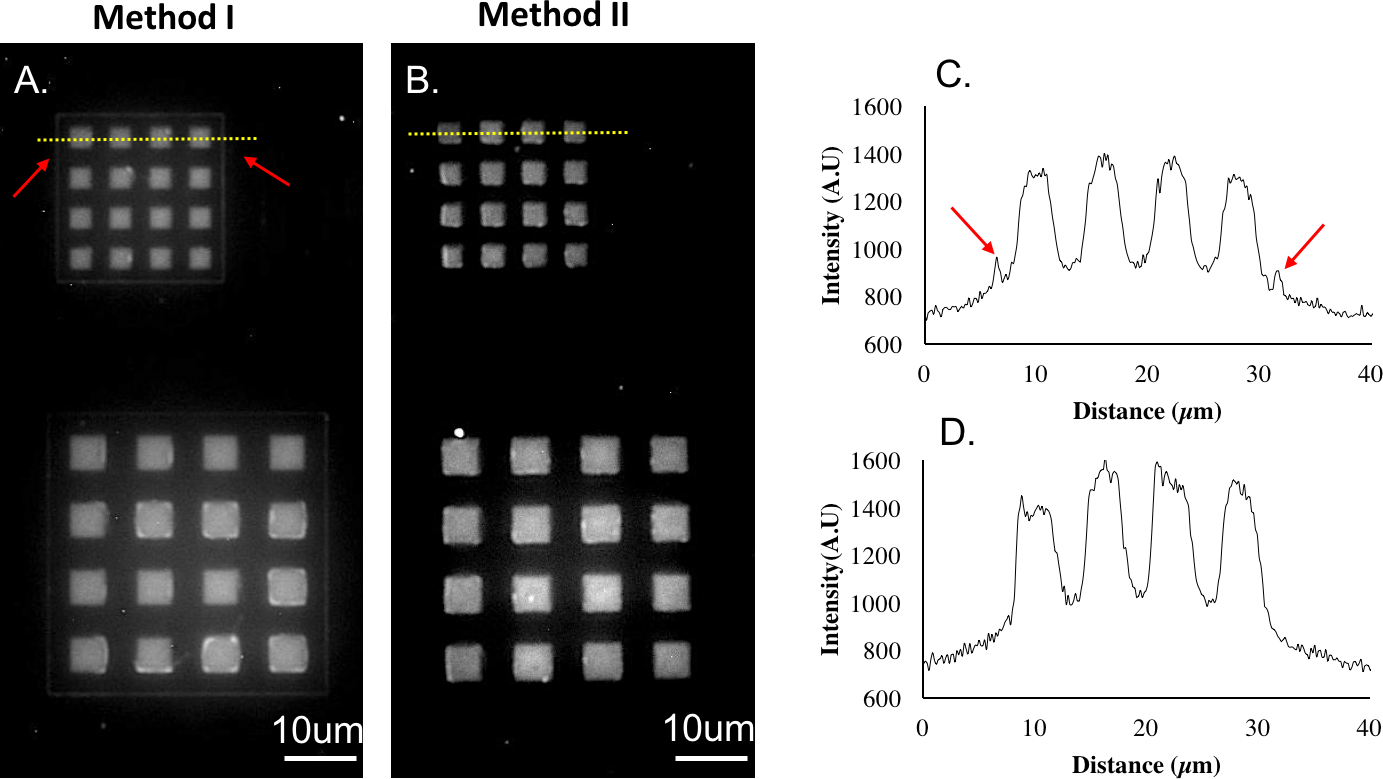
Sidewall coverage depends on how conformal the deposition process is. Biotin microarrays were generated by either Method 1 (A) or Method 2 (B) illustrated in Fig 1. Strep-647 was then used to label biotinylated surfaces. Sidewall conjugation is shown in A) pointed out by red arrows but not in B) due to the higher conformal deposition of LPCVD (B) vs. E-beam evaporation (A). Intensity profiles along the yellow dash lines in A) and B), are shown in C) and D) respectively. Red arrow in C) correspond to the side wall intensity labeled by the red arrows in A). Any misalignment between conjugated regions and wells was too small (<100nm) to measure with our fluorescent microscopy.

As Strep-647 was dissolved in 1X PBS, the germanium layer oxidized and dissolved slowly during the 30min labeling process. The Ge dissolution rate in 0.35% H_2_O_2_ was accelerated if done after a 30min incubation in PBS. Importantly, a 30min incubation in PBS was insufficient to dissolve a 50 nm Ge layer. The germanium did not exhibit appreciable etching until 0.35% H_2_O_2_ was added. This is evidenced by the low background intensity in Fig 3 as the intensity of the unconjugated area was not higher than that of a blank silicon wafer. On the other hand, the Ge surface integrity was less important since a biotin pattern was formed on chip surface during the silane chemistry step. Even if the Ge layer completely dissolves before streptavidin conjugation, streptavidin should only be immobilized on biotin surfaces. Thus, after biotin conjugation, Incubating chips in aqueous solution is not a problem.

All biotinylated regions were successfully conjugated by fluorescent streptavidin. The results for both Ge patterning techniques, Method 1 & 2, are shown in Fig 3A & 3B, respectively. The fluorescent sidewalls in Fig 3A pointed out by red arrows indicate sidewall conjugation (in contrast to Fig 3B, which did not exhibit this). The intensity profiles along yellow dash lines in Fig 3A & 3B are shown in Fig 3C & 3D respectively. Four major peaks represent 4 squares labeled with fluorescent streptavidin. There are two additional little peaks caused by sidewall conjugations on each side of the 4 major peaks, highlighted with red arrows in Fig 3C. These additional intensity peaks have much lower intensity than the major peaks, but still high enough to be distinguished from background, which are however not exhibited in Fig 3D at all. This sidewall conjugation arose because of the non-conformal deposition of Ge evaporation: evaporated ultrathin Ge films (e.g. 50 nm) covered only horizontal surfaces and left vertical sidewalls exposed for biotin conjugation. In contrast, LPCVD Ge films of the same thickness protect vertical sidewalls from surface chemistry.

**Fig 4:**
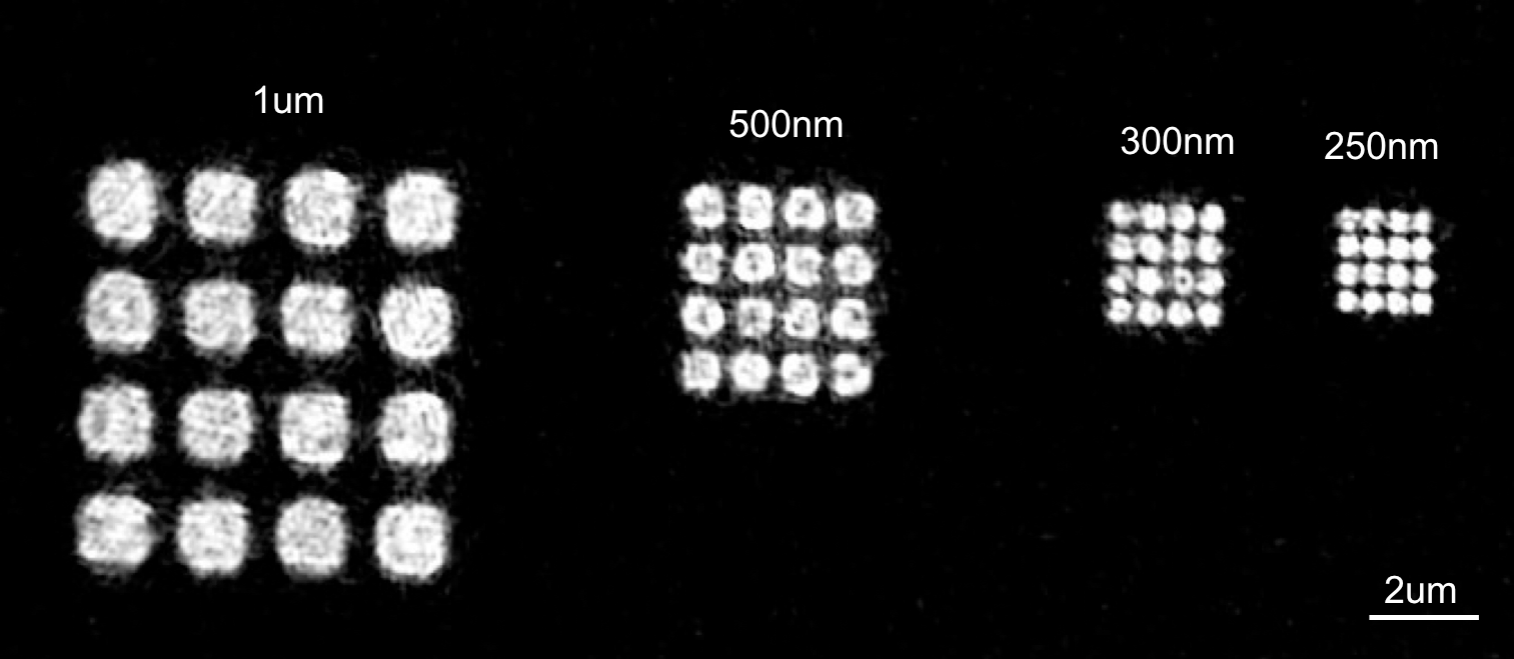
Nanoscale protein patterning. Submicron squares were patterned with biotin and then labeled with strep-647. From left to right the squares have 1 μm, 500 nm, 300 nm and 250 nm sides, respectively; 250 nm is the resolution limit of the lithography tool. The inhomogeneity of fluorescent signal was most likely caused by the nature of super-resolution fluorescent imaging.

The use of modern deep ultraviolet (DUV) lithography enabled nanoscale protein patterning. As shown in Fig 4, all features ranging from 250–1000 nm were successfully resolved and conjugated with biotin. No nonspecific binding was evident. The feature rounding on smaller features was an artifact of submicron lithography.

**Fig 5:**
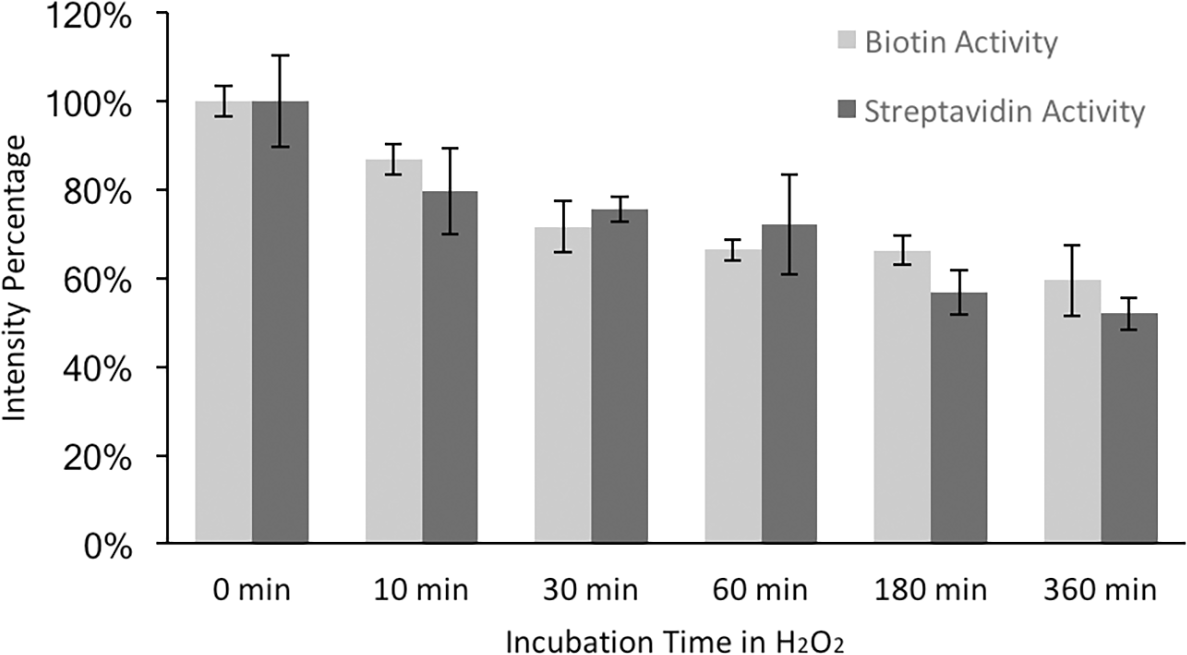
Biotin and streptavidin activity after H_2_O_2_ incubation. Microarrays were fabricated and conjugated with biotin as per Method 2. Streptavidin conjugated chips were obtained by incubating biotin conjugated chips with 100ug/ml non-fluorescent streptavidin solution overnight. Both biotin and streptavidin conjugated chips were then incubated in 0.35% H_2_O_2_ for 10min, 30min, 60min, 3h and 6h at room temperature. After H_2_O_2_ incubation, all chips were blocked in 1%BSA. Biotin chips were then labeled with 10ug/ml Strep-647 while streptavidin chips were labeled with 1ug/ml Biotin-488. Intensity was normalized by using control group (0min) as 100% intensity.

Both biotin and streptavidin could maintain activity after H_2_O_2_ incubation, as shown in Fig 5. The activity was measured in terms of the number of fluorescent biotin or streptavidin molecules bound on streptavidin and biotin surfaces, respectively. Thus, the fluorescent intensity was proportional to the remaining activity of the surface molecule. With 10min 0.35% H_2_O_2_ incubation, both biotin and streptavidin showed ~80% activity. Even after immersing in H_2_O_2_ for 6h, biotin and streptavidin surface still had more than 50% activity remained. Longer incubation was not required for 50nm Ge dissolution; here the longer incubation periods were used to show biotin and streptavidin stability in H_2_O_2_ in case a longer incubation was needed for other applications.

## Conclusion

In this work, we demonstrate the use of germanium sacrificial layers to achieve biomolecule patterning which is compatible with nano/microfabrication, scalable to high resolutions with precise alignment, and ultimately suitable for low cost and high volume manufacturing of biosensors. In addition, the method is applicable to a wide range of biomolecules and surface chemistries. It is of particular utility for surface conjugation chemistries which require organic solvents. Examples include the commonly used primary amine reactive chemical groups for protein labeling, such as N-hydroxysuccinimide ester (NHS), which hydrolyzes quickly in water and the conjugation of proteins onto silicon dioxide surfaces using silane-PEG-NHS. Because both functional groups hydrolyze in water, organic solvents are usually required and a germanium sacrificial layer would be of significant use.

Two processes are reported in this paper, each of which has specific advantages. Method 1 uses evaporation and a lift-off process to pattern the Ge hard mask. This process avoids the high temperature deposition process of Ge and the aggressive etching process in Method 2. For LPCVD processing, all wafers must be cleaned in piranha and hydrogen fluoride (HF). This mandates that there be no organic materials on substrate, which is often not the case for biosensor applications. Since Method 1 uses a lift off technique instead of etching, there is no need to have a stop layer (such as the HTO layer in our case) which is compatible with both the surface chemistry and the etching process. Thus, Method 1 is compatible with low-melting-point biomaterials underneath the Ge layer. We demonstrated nanoscale protein patterning only with Method 2. This is due to lift-off process in Method 1 requiring submicron pillars, which are much harder to resolve than submicron openings with positive photoresists. If an appropriate negative photoresist is available, Method 1 can also be used to resolve nano-features. Furthermore, sidewall functionalization can be achieved and controlled by using a non-conformal Ge deposition process (as in Method 1). On the other hand, the Ge film in Method 2 is much more uniform over the whole surface due to the conformal LPCVD process. It can successfully avoid protein immobilization on sidewalls, which may be preferred for some biosensor applications.

This work employed biotin/streptavidin conjugation to demonstrate the germanium sacrificial proof of concept; both biotin and streptavidin surfaces were stable enough to maintain activity after 0.35% H_2_O_2_ incubation. We also used streptavidin as an example of the method’s use for protein patterning. Of note, streptavidin can be directly used as a cross linker to immobilize biomolecules. For example, after Ge dissolution, a protein can be immobilized on a biotin surface by conjugating a target protein with streptavidin first. Alternatively, streptavidin can be immobilized onto the surface first to capture biotin conjugated proteins onto the surface after Ge dissolution. We believe this method could have wide applicability across a variety of protein patterning applications.
